# Assessment modelling approaches for stocks with spawning components, seasonal and spatial dynamics, and limited resources for data collection

**DOI:** 10.1371/journal.pone.0222472

**Published:** 2019-09-23

**Authors:** Elisabeth Van Beveren, Daniel E. Duplisea, Pablo Brosset, Martin Castonguay

**Affiliations:** Fisheries and Oceans Canada, Institut Maurice-Lamontagne, Mont-Joli, QC, Canada; Aristotle University of Thessaloniki, GREECE

## Abstract

The true spatiotemporal structure of a fish population is often more complex than represented in assessments because movement between spawning components is disregarded and data at the necessary scale are unavailable. This can generate poor advice. We explore the impacts of modelling choices and their associated risks given limited data and lack of biological knowledge on spawning component structure and connectivity. Pseudo-data for an age structured fish population were simulated with two spawning components that experience various levels of connectivity and that might overlap during a certain period but segregate during reproduction. A variety of implicit spatiotemporal and simpler models were fitted to the pseudo-datasets, mimicking different situations of data availability. To reproduce the true stock characteristics, the spatiotemporal models required total catch data disaggregated by spawning component; however, catch-at-age was not as important nor were disaggregated biomass indices to reproduce true dynamics. Even with just 5% connectivity between spawning components, both the spatiotemporal models and simpler alternatives generally overestimated stock biomass. Although bias was smallest when considering one unit population, spawning components might still need to be considered for management and conservation. In such case, the spatiotemporal model was less influenced by ignored connectivity patterns compared to a model focussing on one spawning component only.

## Introduction

Most stock assessments of marine commercial stocks make the dynamic pool assumption [1]: that the stocks are perfectly mixed closed units, without immigration or emigration and seasonal or spatial variation. These assumptions are often flawed, as newer techniques such as data storage tags, genetic markers and otolith microchemistry are now showing that many stocks exhibit a complex spatial and intra-annual structure [2]. Stocks may demonstrate metapopulation structures [3,4] such as organising into separate genetic spawning groups with a rough geographic or phenological identity [5]. Spatial dynamics have been incorporated into some assessments or simulations [6], that can have broadly differing results than assuming a dynamic pool approach. Several studies have shown that not considering some of the important violations of the dynamic pool approach can lead to misspecified assessment models and hence to flawed advice derived from those models [7–13].

However, the true and complex underlying dynamics of a fish stock is commonly not represented by assessment models [6,14]. Often, because of a lack of knowledge on the biological processes involved, it is compelling to ignore these dynamics and to simplify reality. Moreover, if there is a significant exchange of fish between components, it can be necessary to possess high-quality data on which to inform migration rates, although some studies suggest spatial models can be used without for instance any tagging data [15]. Such migration data together with finer-scale spatial and temporal statistics (potentially leading to lower sample sizes and hence more uncertainty) are seldom available. An elevated number of model parameters can also be hard to reliably estimate from typically available data. In addition to such potential overparameterization, suitable models usually result in increased statistical and management complexity.

The limitations of data collection programs with respect to temporal and spatial spawning component structure and fishing dynamics can lead to an assessment framework which fails to capture important aspects of the stock dynamics. Nonetheless, even with science resource constraints, an acceptable assessment framework might still be developed through judicious model assumptions and targeted data collection. For instance, some models which simplify the actual population dynamics might still perform reasonably well under certain circumstances [16,17]. Because there is a clear relationship between data availability and modelling choices, some data may require higher priority than others in order to improve stock evaluation precision and accuracy.

We performed a series of simulations for a theoretical metapopulation in which the two spawning components might overlap spatially for some portion of the year, and segregate during reproduction. In contrast to most simulation studies on fish population dynamics [11,12,18–20], this study is not species-specific although it was inspired by Western Atlantic mackerel (*Scomber scombrus*). We allowed various degrees of connectivity and data availability; for instance catch and biomass index data are sometimes exclusive to a particular spawning component and at other times are from the mixed stock. The simulations tested the trade-off between data availability and model complexity and the ability to produce reasonable estimates from each assessment model. Because in this type of simulation studies a plethora of assumptions needs to be made to which results are intrinsically dependent, we did not intend to estimate the exact performance of each proposed modelling approach. Rather, we aim to get a better overall understanding on the relative effectiveness of each modelling method and on the data requirements associated with them. By doing so we hope to provide generic guidance on how to model a stock with the specified structure and what data to collect to improve the assessment, given limited financial resources.

## Material and methods

### General methodological approach

Simulation testing was done in three steps. First, using a spatiotemporal simulation we refer to as an operating model, pseudo-data was generated for metapopulations of which the spawning components are mixed during half the year and segregated for the other half of the year that includes spawning. Thus the operating model represented the “true” populations. We assumed various migration or connectivity scenarios. Because complete data of the full population are rarely available, we simulated fitting assessment models to only subsets of the operating model output. The performance of model fits was statistically compared to provide guidance on the modelling decision to make under certain data-availability scenarios.

### Operating models

Theoretical or “true” population dynamics (‘pseudo-data’) were simulated in R [21]. Our framework was a statistical catch-at-age model (parameters and equations are in Table 1 and Table A in S1 File, respectively) that included two periods in each year *y* (*p*_1_ and *p*_2_, each lasting six months) and two spawning components (*s*_1_ and *s*_2_). Thus this spatially implicit operating model allowed us to simulate mixed stock components during one period and segregated components during spawning. The system equations for abundance (Table A in S1 File, eq. 1.1a, 1.2a and 1.3a) include a proportion of fish that migrate between stock components (*T*_*s*→!*s*,*y*,*p*,*a*_) which is only applied in *p*_1_, when fish return to their respective spawning grounds. We also included a process error to account for unexplained variations in abundance (e.g. due to migration and natural mortality deviations [22]). Recruitment (Table A in S1 File, eq. 1.1a) takes place for both stocks in the first season and varies around an average value ($\mu_{R_{s}}$) to moderate the number of parameters. Total mortality (*Z*) is the sum of natural (*M*) and fishing (*F*) mortality (Table A in S1 File, eq. 2.2a), with fishing mortality being separable (Table A in S1 File, eq. 2.1a) and each component (combination of *s* an *p*) of annual fishing mortality following a random walk over *y* (Table A in S1 File, eq. 2.3a) and fishing selectivity being knife-edged (maximal at older ages). Spawning stock biomass (*SSB*) at the beginning of each season is given by the product of abundance, weight (*W*) and proportion mature (*P*) (Table A in S1 File, eq. 5.1a).

**Table 1:** Parameters and their definitions by model type (OM = operating model, EM = estimation model).

OM	EM		Definition
spatiotemporal	spatiotemporal	standard	
*Input data*			
*M*_*s*,*y*,*p*,*a*_	*M*_*s*,*y*,*p*,*a*_	*M*_*y*,*a*_	Natural mortality
*P*_*s*,*y*,*p*,*a*_	*P*_*s*,*y*,*p*,*a*_	*P*_*y*,*a*_	Proportion mature
*W*_*s*,*y*,*p*,*a*_	*W*_*s*,*y*,*p*,*a*_	*W*_*y*,*a*_	Weight
*Parameters*			
*N*_*s*,*y*,*p*,*a*_	*N*_*s*,*y*,*p*,*a*_	*N*_*y*,*a*_	Total stock abundance
*F*_*s*,*y*,*p*_	*F*_*s*,*y*,*p*_	*F*_*y*_	Fishing mortality rate
*Sel*_*s*,*p*,*a*_	*Sel*_*s*,*p*,*a*_	*Sel*_*a*_	Fishing selectivity
$\sigma_{I_{s,p}}^{2}$	$\sigma_{I_{s,p}}^{2}$	$\sigma_{I}^{2}$	Index observation error variance
$\sigma_{F_{s,p}}^{2}$	$\sigma_{F_{s,p}}^{2}$	$\sigma_{F_{y}}^{2}$	Annual fishing mortality variance
$\sigma_{{cp}_{s,p}}^{2}$	$\sigma_{{cp}_{s,p}}^{2}$	$\sigma_{cp}^{2}$	Catch proportion observation error variance
$\sigma_{\eta_{s,p,a}}^{2}$	$\sigma_{\eta_{s,p,a}}^{2}$	$\sigma_{\eta_{a}}^{2}$	Process measurement error variance
*q*_*s*,*p*_	*q*_*s*,*p*_	*q*	Index catchability coefficient
$\mu_{R_{s}}$	$\mu_{R_{s}}$	*μ*_*R*_	Average recruitment
*T*_*s*→!*s*,*y*,*p*,*a*_			Transfer coefficient between spawning components

Spawning component, year, period and age are denoted by *s*, *y*, *p* and *a*.

In order to simulate populations, values need to be given to all parameters in Table 1 (see Table B in S1 File), which include starting values for abundance and fishing mortality (only for the first time period). Most parameters (*M*, *P*, *W*, *Sel* and all variances) were determined based on a simplified version of the Northwest Atlantic mackerel stock assessment [23]. We considered 10 age classes (1 to *A*), simulated over 50 years. Starting values of state variables were assumed; fishing mortality was initially presumed to be small and usually increased over time (Figure A in S1 File) and abundance and average recruitment were set so that a wide range of dynamics could be generated (Figure B and C in S1 File). We used the same parameters for both spawning components as a baseline scenario.

Additionally, spawning component connectivity is usually unknown and the key source of uncertainty in simulations thus it influences model performance and choice. Therefore, hypothetical metapopulation dynamics were simulated assuming seven different bilateral connectivity situations (or operating models, Table 2); discrete populations, time- and age- invariant symmetric (5%, 10% and 20%) and asymmetric connectivity (10% versus 20%), time-invariant age-dependent connectivity (following a logistic curve, Figure D in S1 File) and age-invariant density-dependent rates. The latter was calculated as in [17]:

$$T_{s\to !s,y}=\frac{T_{s}^{\prime }}{1+Ae^{-\frac{ln(K)}{B_{s}^{*}}{SSB}_{s,y-1}}}$$

where $T_{s}^{\prime }$ is the maximum total emigration rate out of spawning component s, K is a logistic model parameter and $B_{s}^{*}$ is the x-axis inflection point (with *T*′ = 0.2, *K* = 9, B* = 5.108). Each of these operating models was used for 300 simulations, so that each estimation model could be fitted to 2100 pseudo-populations. Examples of simulated time-series of SSB, fishing mortality, recruitment and density-dependent movement rates are provided as supplementary figures (Figures A, B, C and E in S1 File).

**Table 2:** Operating models (OM) defined by their connectivity (migration) assumptions, as determined by the transfer coefficient (*T*_*s*→!*s*,*y*,*p* = *1*,*a*_).

OM	Scenario	*T*_*s*→!*s*,*y*,*p* = 1,*a*_	*T*_!*s*→*s*,*y*,*p* = 1,*a*_
**1.**	No migration	0	0
**2.**	Symmetric 5%	0.05	0.05
**3.**	Symmetric 10%	0.10	0.10
**4.**	Symmetric 20%	0.20	0.20
**5.**	Asymmetric	0.10	0.20
**6.**	Age-dependent	$\frac{0.2}{1+e^{-(a-4)}}$	$\frac{0.2}{1+e^{-(a-4)}}$
**7.**	Density-dependent	*f*(*SSB*_*s*,*y*,*p* = 1_,*SSB*_!*s*,*y*,*p* = 1_)	*f*(*SSB*_!*s*,*y*,*p* = 1_,*SSB*_*s*,*y*,*p* = 1)_

From each simulation, we generated for both spawning components at each period observations of total catch (Table A in S1 File, eq. 3.2a), catch proportions by age (Table A in S1 File, eq. 3.3a) and an SSB index (Table A in S1 File, eq. 4.1a). To simulate fisheries dependent observations aggregated over periods and/or spawning components, catches (*C*_*s*,*y*,*p*,*a*_) were first summed up in correspondence. For the index we worked with the appropriate abundance estimates; summed up over both spawning components if reflecting the unit population or at spawning if reflecting the year. Catches were calculated with the Baranov catch equation and separated into total catch and catch-at-age proportions, seen as independent observations. Catch proportions were transformed using the continuation-ratio logit approach [24](Table A in S1 File, eq. 3.3a). All three types of simulated data (total catch, catch proportions and the index) had normally-distributed observation error on the transformed scale (Table A in S1 File, eq. 3.4a, 3.5a and 4.2a).

It is impossible to study all conceivable situations and fish stocks, both from a biological and computational point of view, but parameters and data were selected to reflect realistic conditions (i.e., Atlantic mackerel based). Different values will result in different results, but should be of no or inconsequential influence to the general advice this study aims to provide. Simulations were constrained to common situations so that final year biomass did not exceed 10 times the initial biomass. Simulations were also rerun when in at least one year, total catch during mixing (catch for both components combined) was four times higher than the catch of the first spawning component during the spawning season, in order to realistically test a censored spawning component estimation model (see section “Estimation models”).

### Estimation models

**Fig 1:**
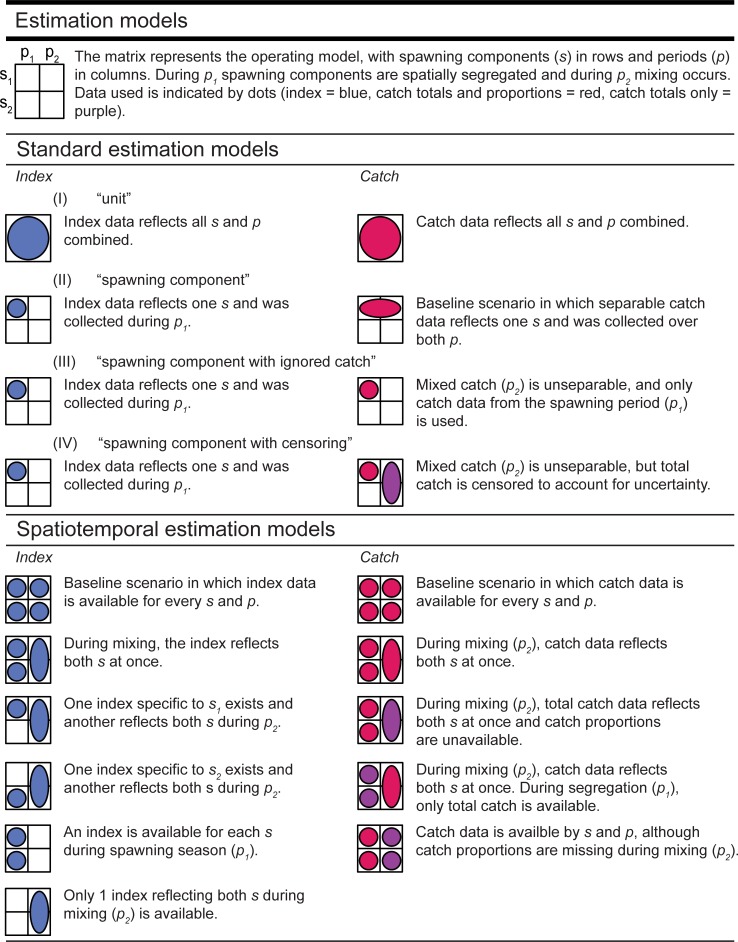
Estimation models (EMs).

Multiple configurations of two types of estimation models were analysed (Fig 1), which do not require tagging data and assume either two discrete spawning components (the discrete spatiotemporal model) or one population (the standard model)(parameters are given in Table 1). Both types were state-space statistical catch-at-age models developed within TMB (Template Model Builder, [25]) that only differed from the operating model in terms of migration (there is no transfer coefficient in the discrete spatiotemporal model, equations are given in Table C in S1 File) or spatiotemporal structure (the standard model assumes a dynamic pool, equations are given in Table D in S1 File). General framework equations and error distributions were otherwise identical. Estimation models were based on observations of total catch, catch-at-age and an SSB index and required typical age-structured data as input (weight, proportion mature and natural mortality). The latter matrices were fixed at their true value, so the ignorance of spatial dynamics and structure is the key driver of estimation error.

The standard model assumed one stock and was based on the Canadian Atlantic mackerel stock assessment [23,26]. Single stock alternatives may be used when the data requirements for a spatiotemporal model are not met, and four configurations could be considered (Fig 1).

- The “unit” estimation model ignores spatial structure and combines all data to represent the entire population (e.g. as in [27–29]).
- The “spawning component” estimation model only reflects one spawning component (here the first) and presumes adequate spawning component-specific catch and index data are available.
- The “spawning component with ignored catch” estimation model presumes that during mixing, not all catches can always be correctly attributed to their spawning component hence are ignored. All observations are taken from the spawning season, when data is certain to only reflect the one spawning component (e.g. as in [30]).
- The “spawning component with censoring” structure is identical to (III), but uses a censored catch approach to incorporate uncertainty around total catches. Catch is censored when the exact value is unknown but some information is available, such as the lower and upper bound [24,26,31,32]. Observation data is still from the spawning season, but total catch is estimated between such bounds; a lower bound determined by the observed catches during the spawning season and an upper bound obtained by adding all catch from the mixed season (similar to [23]).

The discrete spatiotemporal estimation model incorporates two spawning components at once. Contrary to the use of two separate spawning component models, the spatiotemporal model has seasonal time steps, can integrate data reflecting both spawning components and can enforce parameter correspondence (e.g. selectivity or observation errors might be identical). To test minimum data requirements, we tested 30 different estimation models that each leave out or merge different observation data for a certain spawning component or period. To do so we crossed respectively six and five configurations of biomass index and catch data (Table 2). In contrast to SSB indices and catch data (both the total and the proportions), weight-at-age and proportion mature-at-age are fundamental and more easily acquired, and were thus always available for both spawning components and seasons.

### Performance measures

We first verified each model’s stability, defined as the percentage of runs that converged. A run converged when the Hessian and covariance matrices were positive-definite and the maximum gradient component (mgc) did not exceed 0.001 for standard model configurations and 0.01 for spatiotemporal model configurations. The mgc tolerance for the latter was increased because of the high number of parameters and because a simulation study showed good fits even if mgc values were slightly above 0.001 (e.g. as in [33]).

For stable estimation models (> 95% convergence), the converged runs were checked for their ability to correctly estimate SSB and F. For each quantity (*X*), the relative error (*RE*) was calculated by stock, period and year, based on the true and estimated value;

$${RE}_{s,y,p}=\left(\frac{X_{{est}_{s,y,p}}-X_{{true}_{s,y,p}}}{X_{{true}_{s,y,p}}}\right)*100$$

Boxplots integrating all values were used to depict relative error. To assess whether the number of iterations was high enough, we assured that the median *RE*_*SSB*_ had stabilised.

## Results

### Standard estimation models

**Fig 2:**
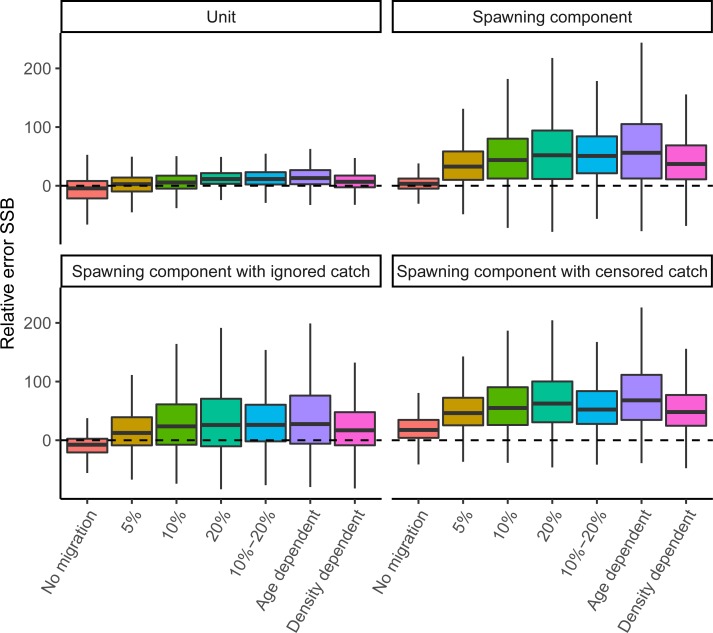
Boxplots of relative error (RE, %) in SSB by the four standard estimations models (panels) based on pseud-data generated by seven operating models (Table 2). The dashed line indicates zero or no error.

**Fig 3:**
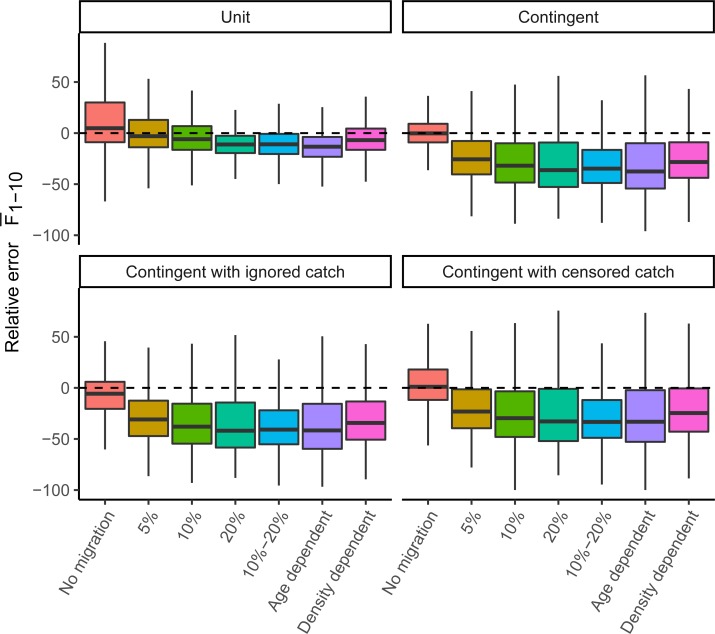
Boxplots of relative error (RE, %) in *F*_1–10_ by the four standard estimations models (panels) based on pseud-data generated by seven operating models (Table 2). The dashed line indicates zero or no error.

Each of the four standard estimation models had good convergence (>95%) and low mgc values (<0.001). When there was no connectivity between spawning components and perfectly disaggregated observations were available, both the unit and spawning component estimated SSB and fishing mortality with < 5% $\bar{RE}$ (RE averaged over all dimensions) (Figs 2 and 3). When modelling a spawning component while ignoring a portion of the catch, the biomass was underestimated (-8% $\bar{RE}$). In contrast, the censored catch model which accounts better for the missing catch overestimated biomass (18% $\bar{RE}$). The larger error is caused by the catch-at-age data, which in both scenarios (ignored and censored catch) was only based on catches from the spawning season (presuming catch during mixing cannot be split). This data is biased because it consists of relatively young individuals compared to the mixing season because of the availability of new age-1 recruits. To fit these biased observations, the model generated more recruits resulting in significant SSB overestimation for the censored model but more correct SSB estimates when catch was partially ignored (the error in total catch and catch-at-age balance one another). When there was no movement, the spawning component model had the lowest interquantile range (on average 17%), followed by the ignored catch (23%), unit (30%) and censored catch scenario (30%).

All patterns of fish connectivity resulted in a high likelihood of overestimating SSB (Fig 2) and correspondingly underestimating fishing mortality (Fig 3), even when both spawning components were modelled as one unit stock. The three model types reflecting one spawning component had the largest difficulty dealing with migration and reacted differently to a net increase or decrease of fish from the spawning component. The net immigration of fish to a spawning component often led to an increased recruitment estimate for that spawning component and a subsequent large overestimate of SSB. When emigration dominated, index catchability (*q*) rather than average recruitment (*μ*_*R*_) was often most biased, which compensated for the loss of fish while causing less bias in SSB and F (Figure F in S1 File). Therefore, the median assessment for a spawning component was more likely to overestimate SSB and underestimate F (Figs 2 and 3). In the presence of migration, the unit model resulted in biomass overestimation (generally <30%) as well, as the generated per-stock observations (index, catch) could, when combined, not be fully described by a model that assumes one homogenous population. Again, biased estimation of catchability and especially average recruitment (parameters *q* and *μ*_*R*_) underlie the observed bias in SSB and F. In all standard models, the catchability and recruitment parameters were most influenced by connectivity, and estimated parameters of observation and process errors were generally unbiased.

### Spatiotemporal estimation models

**Fig 4:**
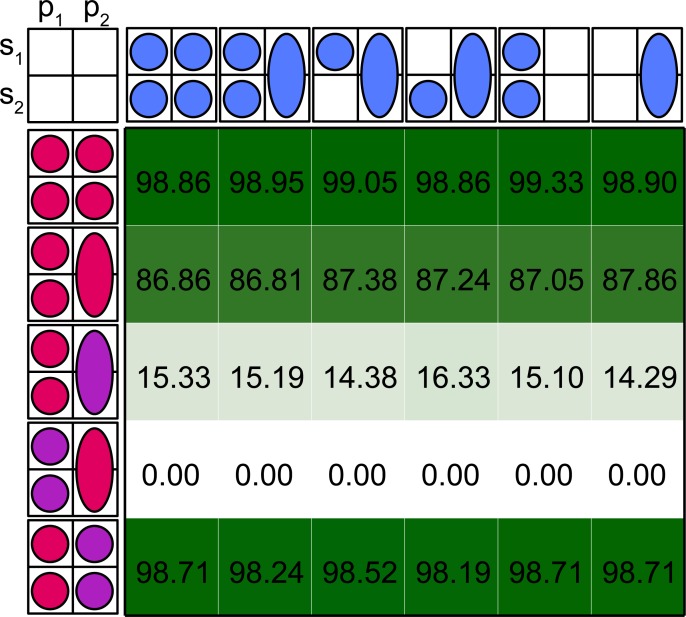
Percentage of convergence of the spatiotemporal model under various scenarios of data availability (see Table 2).

Model stability was dependent on the availability of disaggregated total catch data (Fig 4). When total removals by spawning component were known (row 1 and 5 in Fig 4), a high percentage of models converged (>95%, mgc<0.01), even if catch-at-age proportions were not available during the period of mixing. The inability to separate catches during mixing (row 2 to 4 in Fig 4) precluded convergence too often for the model to be trustworthy during assessments. Additionally, when in these cases convergence was still reached, season specific fishing mortality was often confounded. This model did not have enough information to attribute catch to the correct season so that the random walk on fishing mortality resulted in overly smooth patterns (which is not necessarily visible in aggregated measures of relative error). For these reasons, in the next paragraph we will only discuss the error for scenarios in which total catches are known by spawning component. Unlike total catch information, index data requirements were low, as information is not strictly necessary on a seasonal basis and might even be lumped if reflecting the mixing period. Model convergence was unaffected by the ignorance of connectivity.

**Fig 5:**
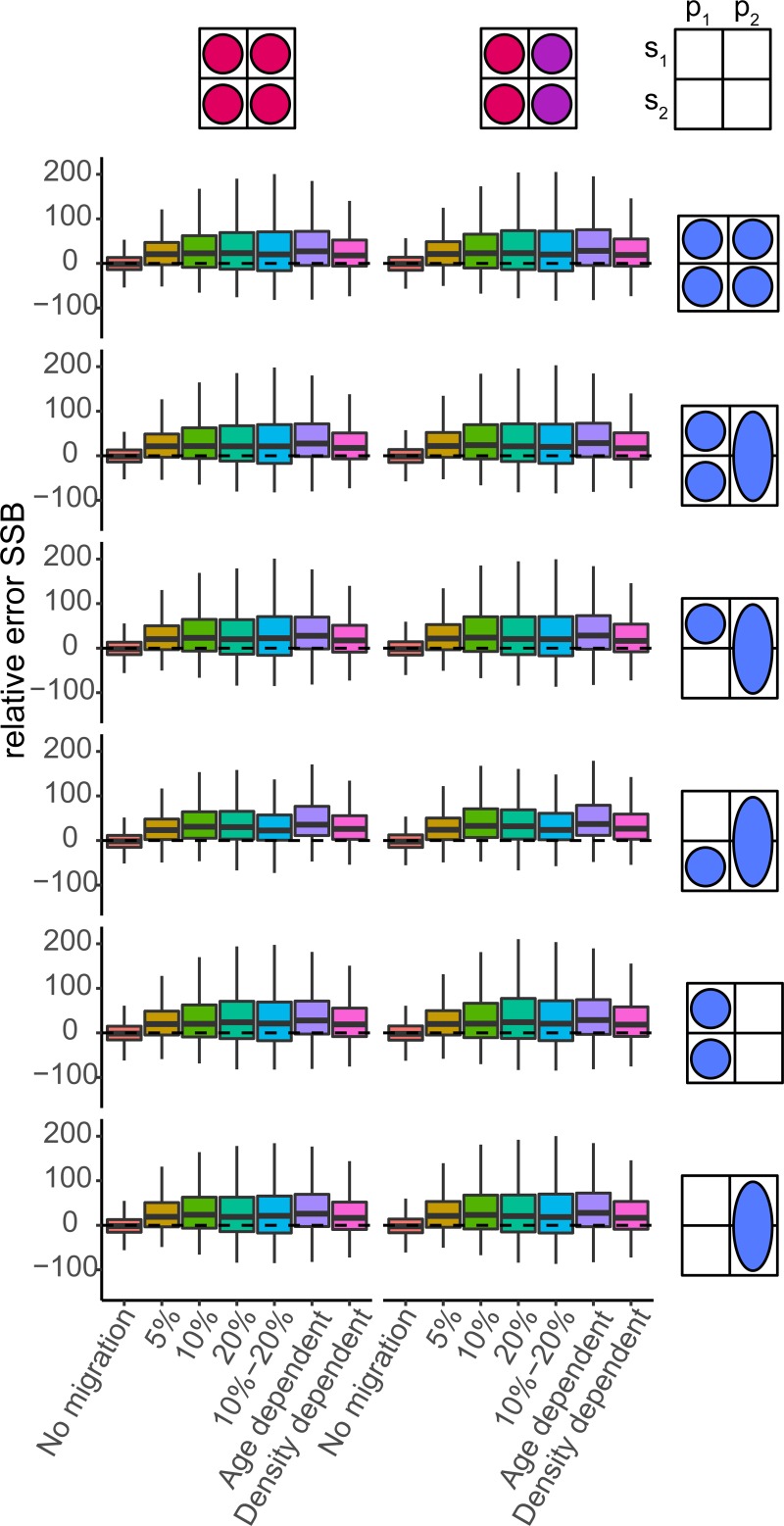
Boxplots of the relative error in SSB for spatiotemporal estimation models with adequate convergence (rows and columns), for each operating model (x-axis).

**Fig 6:**
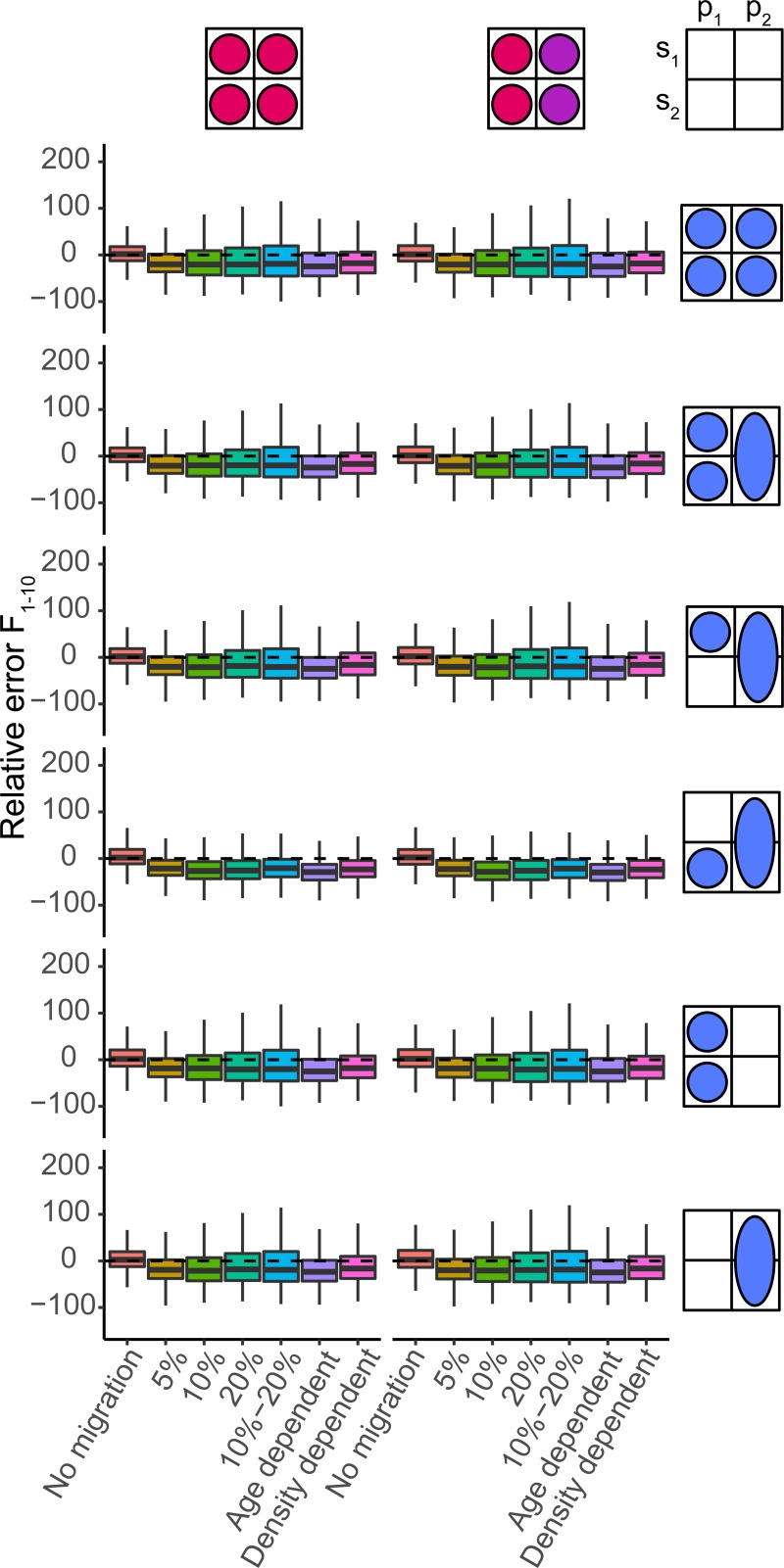
Boxplots of the relative error in F for spatiotemporal estimation models with adequate convergence (rows and columns), for each operating model (x-axis).

When all data are available (Figs 5 and 6, upper left corner) and there is no migration, simulations provided a self-test of the discrete spatiotemporal model [34]. In this baseline scenario, final year estimates of SSB and F_1-10_ (including both stocks and seasons) were unbiased and had relatively low uncertainty as measured by interquantile ranges of -14% to 13% and -12% to 17% respectively.

The lack of fine-scale catch-at-age or index data never diminished model performance, but the ignorance of fish connectivity always resulted in an overestimation of SSB and an underestimation of F (Fig 5 and Fig 6, median RE of on average 19% to 24% and -20% to -22%, respectively, for all connectivity scenarios that were not age-dependent). With the exception of the age dependent movement scenario, the median error did not differ much for all connectivity scenarios, although the interquantile range was wider in cases with stronger fish exchange. The age-dependent migration pattern caused the greatest estimation error (on average 29% for SSB and -25% for F), which is still smaller than the bias caused by all three model types reflecting a spawning component. Analogous to the standard models, the observed bias was related to the erroneous estimation of catchability (*q*) and average recruitment (*μ*_*R*_). Other parameters were estimated without significant bias, except for the catch proportions observation error (*σ*_*cp*_), which increased with intensifying migration to compensate for the generated disturbance in age-structure.

## Discussion

Stock assessment scientists must often contend with a lack of data or biological knowledge of stock structure and mixing of assessed populations. Our goal was to provide guidance on assessment approaches and/or data collection to produce the best scientific advice given these unknowns. We created a simulation based on the simplest situation assuming a stock with two spawning components that overlap during six months but segregate during spawning, and with different levels of exchange of group membership. The observations and guidance provided are generalized and provide some understanding of where biases lay in assessment estimates in more complex but qualitatively similar ignorance situations.

In the trivial situation when spawning components are unconnected biologically and are fished separately with spawning component-specific data reporting, the unit and spawning component models performed well without bias. A cornerstone of these standard stock assessment models is often the assumption that fishing mortality rates are constant within a year (a consequence of the Baranov catch equation), which only rarely occurs in reality and was untrue in our simulations as well. This violation of temporal homogeneity did not however significantly affect the performance of these model types. There is thus no strong incentive to acquire data or model the population dynamics at a finer time scale when fishing intensity changes over the year. Our study therefore agrees with Liu [35], who also indicated that the Baranov catch equation can, with some exceptions, provide a reasonable approximation even when its constant fishing mortality assumption is violated.

Poor performance of the unit and spawning component models was predominantly triggered by connectivity ignorance rather than seasonal patterns of fishing. Even the unit model which lumped both components was somewhat sensitive to connectivity, even though fish exchange promoted homogenisation. This shows that the dynamic pool assumption can lead to biased estimates of the important quantities of SSB and F when there are mixed fisheries. The unit model was the least biased estimator for SSB and F and did not require a full suite of spawning component data, but might not be the optimal assessment model choice. The unit model could possibly lead to collapse of spawning components [8,36], because fishing pressure cannot be exerted in proportion to the relative size of the spawning components, resulting in the overexploitation of at least one component. The loss of spawning components in a metapopulation context can lead to a loss of overall stability and resilience to ecosystem and environmental changes [37–39]. The unit population approach might also be suboptimal when spawning components are in different management units (or jurisdictions) so that modelling them separately might be required for practical and logistic reasons.

Modelling separate spawning components might be preferred if data are or can be made available by component, even though it might produce greater inaccuracy (as in [10]), even when ignored connectivity rates are low. Over all our connectivity simulations, SSB was usually overestimated by more than 30%, which indicates the importance of gathering additional data and knowledge on migration patterns and strength, or the need for case-specific simulation studies to determine more precisely the potential inaccuracy and the effect on stock management (e.g. [15]). For instance, in a spawning component model the bias introduced by ignoring connectivity is positively correlated with the dissimilarity between the dynamics of the spawning components [16,40]. This might explain the relatively large error and error range observed here, as our simulations spanned a wide range of possible population dynamics, despite spawning component parameters being identical. Nonetheless, neglecting spawning component dependence generally resulted in the overestimation of SSB and underestimation of F (as found by [16]), which might lead to overexploitation, in agreement with [12].

When spawning components overlap during one season, the application of a spawning component model might also be questionable when landed fish cannot be attributed to their component (e.g. Northwest Atlantic mackerel [41]) or only imprecisely so (e.g. herring west of the British Isles [10]). Surprisingly, ignoring this proportion of the mixed catches improved the estimates, as the underestimation of SSB resulting from catch underreporting (see e.g. [42]) partially balanced the overestimation of SSB caused by unaccounted for migration. Hence, the scales of ignored catch (at most 4 times the spawning season catch) appeared less important than those of migration. Integrating catch uncertainty by means of censoring approached the results of simulations in which catch data could be perfectly split, so that this method did not improve estimates relative to the scenario in which catches from the mixing season were completely ignored. These results do not warrant omitting part of the total catch, but rather stress the unexpected behaviour of complex models when several mismatches between the truth and the assumed reality occur.

As all standard models have some issues, we also investigated a compound model which bridges these simpler models and complex data-demanding models. In contrast to the complex spatially explicit models, the investigated spatially implicit model might perform still reasonably well without hard to get movement data and a mismatch between management areas and biological entities is avoided [18]. Although our discrete spatiotemporal model is essentially a doubling of the spawning component model with a seasonal time step, there are several reasons to potentially favour its use if sufficiently fine-scale data would be available. The integrated seasonality could in part buffer against the lack of knowledge on movement, as smaller time steps might allow for a more accurate accounting of F and SSB. For instance, age specific migration might be captured within seasonal rather than annual fishery selectivity curves [43,44]. Likewise, seasonal process error might better capture migration-induced irregularities than an annual matrix. Indeed, the estimation error in this model due to migration was notably smaller relative to the spawning component models, albeit still higher than for the unit model. The spatiotemporal model also has the advantage that all spawning components are considered at once so that biological and observational parameters can be harmonized, aggregated data can be used and it is a stepping stone towards a model that explicitly integrates fish exchange.

We provided information on the minimal data requirements for fitting such spatiotemporal models by analysing various data availability scenarios. We showed that the spatiotemporal model can only be stable if total catches can be separated by stock and season, although mixed total catch did not completely preclude their use. Additionally, care might also be needed when using mixed catch, as preliminary analyses showed that in certain cases seasonal fishing mortality rates were confounded. This corroborates the findings of other studies based on different models, which indicated the necessity to identify the origin of the fish in the catches [10,45]. Nonetheless, stock dynamics could still be reproduced when catch-at-age data were unavailable during the mixing season and as little as a single biomass index reflecting both spawning components exists. With a small amount of data, model performance needs deeper scrutiny. These results suggest that for spatiotemporal model development, effort should first be directed towards the separation of catches, as without this information little improvement can be made in the representation of true stock dynamics.

Simulation testing is a very flexible and powerful tool to show general results as we have done here, but the full complexity of population dynamics can never be captured [46,47]. Many assumptions were made and it is impossible to test the sensitivity of the results to all of them. For instance, simulations were made for a population with two spawning components composed of 10 age classes, using a fixed set of parameters identical for both spawning components and working with a non-age-specific biomass index. By assuming a less complex ‘truth’, some results might appear overly optimistic. In reality, some observed data might be biased or have a larger observation error, potentially lowering the performance of all estimation models. For example, the spatiotemporal model might have difficulty converging when seasonal data are somewhat conflicting. Natural mortality is not usually known, especially at age and between years, which should particularly affect the censored spawning component model, as previous studies have indicated its sensitivity to this statistic [26,48]. Also, if spawning components are subject to or characterised by dissimilar selectivity, process or observation variances, the underlying assumptions of the unit model would be further violated and its performance might potentially be diminished. The simplicity of the migration patterns is unlikely to occur in the real world as well, but because all scenarios considered (age-structured or not) were biased in a very similar way, more complex and realistic estimates would probably have provided the same conclusions.

Despite these limitations, we were able to shed light on the advantages and disadvantages of different assessment approaches when facing common problems related to the spatial and temporal dynamics of spawning components as well as imperfect data. Although the unit model was the most accurate, we know, as argued earlier, that it is unlikely to provide proper management of the spawning components. When data availability is minimal, the priority should be the development of spawning component specific indices as well as methods to estimate catch proportions so that a spawning component model might be used. When applying spawning component models in this study, problems arising from mixed spawning components in catches were inferior to the ignorance of small movement rates and the interaction of both uncertainties resulted in lower but more unpredictable bias. Although bias might be significantly reduced by acquiring detailed movement data, the temporal refinement of model data (e.g., weight and maturity data) might be easier in practice and can result in a compound model that is more robust to this lack of knowledge. Still, none of the investigated models could fully buffer against the bias generated by overlooking various levels and types of connectivity. Even at low migration rates, there might be significant consequences for our perception and the management of stocks, and there is no obvious workaround. It is evident that the gathering of new knowledge is always advantageous for the assessment process, but with restricted possibilities, the focus should not only be on model choices, but also on the development of management strategies that are robust to uncertainty in connectivity and mixing rates.

## Supporting information

S1 File Model details. (DOCX)
