# Supplementary material for: Assessment modelling approaches for stocks with spawning components, seasonal and spatial dynamics, and limited resources for data collection
Source: PLoS One. 2019 Sep 23;14(9):e0222472. doi: 10.1371/journal.pone.0222472 (PMC6756546; doi:10.1371/journal.pone.0222472)
Supplement: S1 File — (DOCX) [file pone.0222472.s001.docx]

**PLoS ONE**

# *Supplementary Information*

**Assessment Modelling Approaches for Stocks with Spawning Components, Seasonal and Spatial Dynamics, and Limited Resources for Data Collection**

Elisabeth Van Beveren^1^, Daniel E. Duplisea^1^, Pablo Brosset^1^, Martin Castonguay^1^

^1^Fisheries and Oceans Canada, Institut Maurice-Lamontagne, 850 Route de la Mer, Mont-Joli, G5H 3Z4, QC, Canada

*** Corresponding author**: e-mail: [elisabeth.vanbeveren@dfo-mpo.gc.ca](mailto:elisabeth.vanbeveren@dfo-mpo.gc.ca)

Table A. Operating model equations (a = age, y = year, p = period, o = observed, W = weight, P = proportion mature, N = abundance, M = natural mortality, F = fishing mortality, Z = total mortality, C = catch, CT = total annual catch, CP = catch proportions, Sel = selectivity, I = index, ts = timing of the survey, crl = continuation-ratio logit transformation, MVN = multivariate normal). Note that for cohort abundance equations we use time (t) as a combination of year and period to easier denote transitions to the next time slot.

| **Operating model equations** | |  |
| --- | --- | --- |
| *Parameter* | *Formula* | *No.* |
| Cohort abundance | $N_{s,t,1}=\mu_{R_{s}}e^{\varepsilon_{s,t,1}^{\eta}}$ | 1.1a |
|  | $N_{s,t1, stocksts of ions are:th two contingents ()sensored catch used during the Canadian assessment of Western Atlantic mackerel (,a}=\left[ \left( 1-T_{s\to!s,t,a} \right)N_{s,t-1,a-1}e^{-Z_{s,t-1,a-1}}+T_{!s\to s,t,a}N_{!s,t-1,a-1}e^{-Z_{!s,t-1,a-1}} \right]e^{\varepsilon_{s,t,a}^{\eta}}$ | 1.2a |
|  | $N_{s,t,A}=\left[ \left( 1-T_{s\to!s,t,a} \right)N_{s,t-1,A-1}e^{-Z_{s,t-1,A-1}}+T_{!s\to s,t,a}N_{!s,t-1,A-1}e^{-Z_{!s,t-1,A-1}}+\left( 1-T_{s\to!s,t,a} \right)N_{s,t-1,A}e^{-Z_{s,t-1,A}}+T_{!s\to s,t,a}N_{!s,t-1,A}e^{-Z_{!s,t-1,A}} \right]e^{\varepsilon_{s,t,A}^{\eta}}$ | 1.3a |
|  | $\varepsilon_{s,t,a}^{\eta} \sim MVN(0,\sigma_{\eta_{s,p,a}}^{2})$ | 1.4a |
| Mortality rates | $F_{s,y,p,a}={Sel}_{s,p,a}F_{s,y,p}$ | 2.1a |
|  | $Z_{s,y,p,a}=F_{s,y,p,a}+M_{s,y,p,a}$ | 2.2a |
|  | $F_{s,y,p}=F_{s,y-1,p}e^{\varepsilon_{s,y,p}^{F}}$ | 2.3a |
|  | $\varepsilon_{s,y,p}^{F} \sim N(0,\sigma_{F_{s,p}}^{2})$ | 2.4a |
| Catch | $C_{s,y,p,a}\text{=}N_{s,y,p,a}\frac{F_{s,y,p,a}}{Z_{s,y,p,a}}\left[ 1-e^{-Z_{s,y,p,a}} \right]$ | 3.1a |
|  | ${CT}_{s,y,p}=\left( \sum_{a=1}^{A} C_{s,y,p,a}W_{s,y,p,a} \right)e^{\varepsilon_{s,y,p}^{ct}}$ | 3.2a |
|  | ${CP}_{s,y,p,a}=\frac{C_{s,y,p,a}}{\sum_{a=1}^{A} C_{s,y,p,a}}$ | 3.3a |
|  | $X_{s,y,p,a}=crl({CP}_{s,y,p,a}) e^{\varepsilon_{s,y,p,a}^{cp}}$ | 3.3a |
|  | $\varepsilon_{s,y,p}^{ct} \sim N(0,0.01)$ | 3.4a |
|  | $\varepsilon_{s,y,p,a}^{cp} \sim N(0,\sigma_{{cp}_{s,p}}^{2})$ | 3.5a |
| Survey index | $I_{s,y,p}=\left( \sum_{a=1}^{A} q_{s,p}N_{s,y,p,a}e\left( -Z_{s,y,p,a}{ts}_{s,p} \right)W_{s,y,p,a}P_{s,y,p,a} \right)e^{\varepsilon_{s,y,p}^{I}}$ | 4.1a |
|  | $\varepsilon_{s,y,p}^{I} \sim N(0,\sigma_{I_{s,p}}^{2})$ |  |
| Spawning Stock Biomass | ${SSB}_{s,y,p}=\sum_{a=1}^{A} N_{s,y,p,a}W_{s,y,p,a}P_{s,y,p,a}$ | 5.1a |

|  |  | Value | | | | | | | | | | | | | | | | | | | | |
| --- | --- | --- | --- | --- | --- | --- | --- | --- | --- | --- | --- | --- | --- | --- | --- | --- | --- | --- | --- | --- | --- | --- |
| *period* |  | $p_{1}$ | | | | | | | | | | | $p_{2}$ | | | | | | | | | |
| *age* |  | *1* | *2* | | *3* | *4* | *5* | *6* | *7* | *8* | *9* | *10* | *1* | *2* | *3* | *4* | *5* | *6* | *7* | *8* | *9* | *10* |
| $\boldsymbol{M}_{\boldsymbol{s,p,a}}$ | $s_{1}$ | 0.1 | 0.1 | | 0.1 | 0.1 | 0.1 | 0.1 | 0.1 | 0.1 | 0.1 | 0.1 | 0.1 | 0.1 | 0.1 | 0.1 | 0.1 | 0.1 | 0.1 | 0.1 | 0.1 | 0.1 |
|  | $s_{2}$ | 0.1 | 0.1 | | 0.1 | 0.1 | 0.1 | 0.1 | 0.1 | 0.1 | 0.1 | 0.1 | 0.1 | 0.1 | 0.1 | 0.1 | 0.1 | 0.1 | 0.1 | 0.1 | 0.1 | 0.1 |
| $\boldsymbol{P}_{\boldsymbol{s,p,a}}$ | $s_{1}$ | 0.2 | 0.8 | | 0.94 | 0.98 | 1 | 1 | 1 | 1 | 1 | 1 | 0.2 | 0.8 | 0.94 | 0.98 | 1 | 1 | 1 | 1 | 1 | 1 |
|  | $s_{2}$ | 0.2 | 0.8 | | 0.94 | 0.98 | 1 | 1 | 1 | 1 | 1 | 1 | 0.2 | 0.8 | 0.94 | 0.98 | 1 | 1 | 1 | 1 | 1 | 1 |
| $\boldsymbol{W}_{\boldsymbol{s,p,a}}$ | $s_{1}$ | 0.1 | 0.2 | | 0.3 | 0.4 | 0.5 | 0.6 | 0.7 | 0.7 | 0.7 | 0.7 | 0.1 | 0.2 | 0.3 | 0.4 | 0.5 | 0.6 | 0.7 | 0.7 | 0.7 | 0.7 |
|  | $s_{2}$ | 0.1 | 0.2 | | 0.3 | 0.4 | 0.5 | 0.6 | 0.7 | 0.7 | 0.7 | 0.7 | 0.1 | 0.2 | 0.3 | 0.4 | 0.5 | 0.6 | 0.7 | 0.7 | 0.7 | 0.7 |
| $\boldsymbol{log}\boldsymbol{N}_{\boldsymbol{s,y=1,p=1,a}}$ | $s_{1}$ | 15 | 15.56 | | 16.11 | 16.67 | 17.22 | 17.78 | 18.33 | 18.89 | 19.44 | 20 | 15 | 15.56 | 16.11 | 16.67 | 17.22 | 17.78 | 18.33 | 18.89 | 19.44 | 20 |
|  | $s_{2}$ | 15 | 15.56 | | 16.11 | 16.67 | 17.22 | 17.78 | 18.33 | 18.89 | 19.44 | 20 | 15 | 15.56 | 16.11 | 16.67 | 17.22 | 17.78 | 18.33 | 18.89 | 19.44 | 20 |
| $\boldsymbol{Sel}_{\boldsymbol{s,p,a}}$ | $s_{1}$ | 0.1 | 0.4 | | 0.8 | 1 | 1 | 1 | 1 | 1 | 1 | 1 | 0.1 | 0.4 | 0.8 | 1 | 1 | 1 | 1 | 1 | 1 | 1 |
|  | $s_{2}$ | 0.1 | 0.4 | | 0.8 | 1 | 1 | 1 | 1 | 1 | 1 | 1 | 0.1 | 0.4 | 0.8 | 1 | 1 | 1 | 1 | 1 | 1 | 1 |
| $\boldsymbol{\sigma}_{\boldsymbol{\eta}_{\boldsymbol{s,p,a}}}^{\boldsymbol{2}}$ | $s_{1}$ | 0.4 | 0.2 | | 0.2 | 0.2 | 0.2 | 0.2 | 0.2 | 0.2 | 0.2 | 0.2 | 0.4 | 0.2 | 0.2 | 0.2 | 0.2 | 0.2 | 0.2 | 0.2 | 0.2 | 0.2 |
|  | $s_{2}$ | 0.4 | 0.2 | | 0.2 | 0.2 | 0.2 | 0.2 | 0.2 | 0.2 | 0.2 | 0.2 | 0.4 | 0.2 | 0.2 | 0.2 | 0.2 | 0.2 | 0.2 | 0.2 | 0.2 | 0.2 |
| *period* |  | $p_{1}$ | | $p_{2}$ | | | | | | | | | | | | | | | | | | |
| $\boldsymbol{F}_{\boldsymbol{s,y=1,p=1}}$ | $s_{1}$ | 0.05 | | - | | | | | | | | | | | | | | | | | | |
|  | $s_{2}$ | 0.05 | | - | | | | | | | | | | | | | | | | | | |
| $\boldsymbol{\sigma}_{\boldsymbol{I}_{\boldsymbol{s,p}}}^{\boldsymbol{2}}$ | $s_{1}$ | 0.5 | | 0.5 | | | | | | | | | | | | | | | | | | |
|  | $s_{2}$ | 0.5 | | 0.5 | | | | | | | | | | | | | | | | | | |
| $\boldsymbol{\sigma}_{\boldsymbol{F}_{\boldsymbol{s,p}}}^{\boldsymbol{2}}$ | $s_{1}$ | 0.3 | | 0.3 | | | | | | | | | | | | | | | | | | |
|  | $s_{2}$ | 0.3 | | 0.3 | | | | | | | | | | | | | | | | | | |
| $\boldsymbol{\sigma}_{\boldsymbol{cp}_{\boldsymbol{s,p}}}^{\boldsymbol{2}}$ | $s_{1}$ | 0.1 | | 0.1 | | | | | | | | | | | | | | | | | | |
|  | $s_{2}$ | 0.1 | | 0.1 | | | | | | | | | | | | | | | | | | |
| $\boldsymbol{q}_{\boldsymbol{s,p}}$ | $s_{1}$ | 1 | | 1 | | | | | | | | | | | | | | | | | | |
|  | $s_{2}$ | 1 | | 1 | | | | | | | | | | | | | | | | | | |
| $\boldsymbol{log\mu}_{\boldsymbol{R}_{\boldsymbol{s}}}$ | $s_{1}$ | 20 | | - | | | | | | | | | | | | | | | | | | |
|  | $s_{1}$ | 20 | | - | | | | | | | | | | | | | | | | | | |
| $\boldsymbol{T}_{\boldsymbol{s\to!s,y,p=1,a}}$ | $s_{1}$ | see Table 2 | | - | | | | | | | | | | | | | | | | | | |
|  | $s_{1}$ | see Table 2 | | - | | | | | | | | | | | | | | | | | | |

Table B. Operating model values used to simulate population dynamics.

Table C. Equations from the spatiotemporal estimation model (a = age, y = year, o = observed, W = weight, P = proportion mature, N = abundance, M = natural mortality, F = fishing mortality, Z = total mortality, C = catch, CT = total annual catch, CP = catch proportions, Sel = selectivity, I = index, ts = timing of the survey, crl = continuation-ratio logit transformation, MVN = multivariate normal). Note that for cohort abundance equations we use time (t) as a combination of year and period to easier denote transitions to the next time slot.

| **Estimation model equations (spatiotemporal)** | |  |
| --- | --- | --- |
| *Parameter* | *Formula* | *No.* |
| Cohort abundance | $N_{s,t,1}=\mu_{R_{s}}e^{\varepsilon_{s,t,1}^{\eta}}$ | 1.1b |
|  | ${N_{s,t,a}=N}_{s,t-1,a-1}e^{-Z_{s,t-1,a-1}+\varepsilon_{s,t,a}^{\eta}}$ | 1.2b |
|  | ${N_{s,t,A}=[N}_{s,t-1,A-1}e^{-Z_{s,t-1,A-1}}+N_{s,t-1,A}e^{-Z_{s,t-1,A}}]e^{\varepsilon_{s,t,A}^{\eta}}$ | 1.3b |
|  | $\varepsilon_{s,t,a}^{\eta} \sim MVN(0,\sigma_{\eta_{s,p,a}}^{2})$ | 1.4b |
| Mortality rates | $F_{s,y,p,a}={Sel}_{s,p,a}F_{s,y,p}$ | 2.1b |
|  | $Z_{s,y,p,a}=F_{s,y,p,a}+M_{s,y,p,a}$ | 2.2b |
|  | $F_{s,y,p}=F_{s,y-1,p}e^{\varepsilon_{s,y,p}^{F}}$ | 2.3b |
|  | $\varepsilon_{s,y,p}^{F} \sim N(0,\sigma_{F_{s,p}}^{2})$ | 2.4b |
| Catch | $C_{s,y,p,a}\text{=}N_{s,y,p,a}\frac{F_{s,y,p,a}}{Z_{s,y,p,a}}\left[ 1-e^{-Z_{s,y,p,a}} \right]$ | 3.1b |
|  | ${CT}_{s,y,p}=\sum_{a=1}^{A} C_{s,y,p,a}W_{s,y,p,a}$ | 3.2b |
|  | ${CP}_{s,y,p,a}=\frac{C_{s,y,p,a}}{\sum_{a=1}^{A} C_{s,y,p,a}}$ | 3.3b |
|  | $X_{s,y,p,a}=crl({CP}_{s,y,p,a})$ | 3.3b |
|  | $l\left( \left\{ X_{o_{s,y,p,a}} \right\} \vert\theta\right)=\sum_{\boldsymbol{a}=\boldsymbol{1}}^{\boldsymbol{A}-\boldsymbol{1}} \sum_{Y=1}^{Y} log\left[ \varphi_{N}\left( \frac{X_{o_{s,y,p,a}}-X_{s,y,p,a}}{\sigma_{{cp}_{s,p}}} \right) \right]$ | 3.4b |
|  | $l\left( \left\{ {CT}_{o_{s,y,p}} \right\} \vert\theta\right)=\sum_{\boldsymbol{a}=\boldsymbol{1}}^{\boldsymbol{A}} \sum_{Y=1}^{Y} log\left[ \varphi_{N}\left( \frac{{CT}_{o_{s,y,p}}-{CT}_{s,y,p}}{0.01} \right) \right]$ | 3.5b |
| Survey index | $I_{s,y,p}=\sum_{a=1}^{A} q_{s,p}N_{s,y,p,a}e\left( -Z_{s,y,p,a}{ts}_{s,p} \right)W_{s,y,p,a}P_{s,y,p,a}$ | 4.1b |
|  | $l\left( \left\{ I_{o_{s,y,p}} \right\} \vert\theta\right)=\sum_{\boldsymbol{a}=\boldsymbol{1}}^{\boldsymbol{A}} \sum_{Y=1}^{Y} log\left[ \varphi_{N}\left( \frac{I_{o_{s,y,p}}-I_{s,y,p}}{\sigma_{I_{s,p}}} \right) \right]$ | 4.2b |
| Spawning Stock Biomass | ${SSB}_{s,y,p}=\sum_{a=1}^{A} N_{s,y,p,a}W_{s,y,p,a}P_{s,y,p,a}$ | 5.1b |

Table D. Equations from the standard estimation model (a = age, y = year, o = observed, W = weight, P = proportion mature, N = abundance, M = natural mortality, F = fishing mortality, Z = total mortality, C = catch, CT = total annual catch, CP = catch proportions, Sel = selectivity, I = index, ts = timing of the survey, crl = continuation-ratio logit transformation, U = upper catch limit, MVN = multivariate normal). For eq. 3.5 two different likelihoods can be used (censored or not). Note that for cohort abundance equations we use time (t) as a combination of year and period to easier denote transitions to the next time slot.

| **Estimation model equations (standard)** | |  |
| --- | --- | --- |
| *Parameter* | *Formula* | No. |
| Cohort abundance | $N_{1,y}=\mu_{R_{s}}e^{\varepsilon_{1,y}^{\eta}}$ | 1.1c |
|  | ${N_{a,y}=N}_{a-1,y-1}e^{-Z_{a-1,y-1}+\varepsilon_{a,y}^{\eta}}$ | 1.2c |
|  | ${N_{A,y}=[N}_{A-1,y-1}e^{-Z_{A-1,y-1}}+N_{A,y-1}e^{-Z_{A,y-1}}]e^{\varepsilon_{A,y}^{\eta}}$ | 1.3c |
|  | $\varepsilon_{a,y}^{\eta} \sim MVN(0,\sigma_{\eta_{a}}^{2})$ | 1.4c |
| Mortality rates | $F_{a,y}={Sel}_{a}F_{y}$ | 2.1c |
|  | $Z_{a,y}=F_{a,y}+M_{a,y}$ | 2.2c |
|  | $F_{y}=F_{y-1}e^{\varepsilon_{y}^{F}}$ | 2.3c |
|  | $\varepsilon_{y}^{F} \sim N(0,\sigma_{F}^{2})$ | 2.4c |
| Catch | $C_{a,y}\text{=}N_{a,y}\frac{F_{a,y}}{Z_{a,y}}\left[ 1-e^{-Z_{a,y}} \right]$ | 3.1c |
|  | ${CT}_{y}=\sum_{a=1}^{A} C_{a,y}W_{a,y}$ | 3.2c |
|  | ${CP}_{a,y}=\frac{C_{a,y}}{\sum_{a=1}^{A} C_{a,y}}$ | 3.3c |
|  | $X_{a,y}=crl({CP}_{a,y})$ | 3.3c |
|  | $l\left( \left\{ X_{o_{a,y}} \right\} \vert\theta\right)=\sum_{\boldsymbol{a}=\mathbf{1}}^{\boldsymbol{A}-\mathbf{1}} \sum_{Y=1}^{Y} log\left[ \varphi_{N}\left( \frac{X_{o_{a,y}}-X_{a,y}}{\sigma_{cp}} \right) \right]$ | 3.4c |
| *uncensored* | $l\left( \left\{ {CT}_{o_{y}} \right\} \vert\theta\right)=\sum_{\boldsymbol{a}=\mathbf{1}}^{\boldsymbol{A}} \sum_{Y=1}^{Y} log\left[ \varphi_{N}\left( \frac{{CT}_{o_{y}}-{CT}_{y}}{0.01} \right) \right]$ | 3.5c (1) |
| *censored* | $l\left( \left\{ {CT}_{o1},\ldots,{CT}_{oY} \right\}\vert\theta\right)=\sum_{y=1}^{Y} log\left\{ \phi_{N}\left[ \frac{log\left( {{CT}_{{oU}_{y}}}/{{CT}_{y}} \right)}{0.01} \right]-\phi_{N}\left[ \frac{log\left( {{CT}_{{oL}_{y}}}/{{CT}_{y}} \right)}{0.01} \right] \right\}$ | 3.5c (2) |
| Survey index | $I_{y}=\sum_{a=1}^{A} qN_{a,y}e\left( -Z_{a,y}\mathrm{ts} \right)W_{a,y}P_{a,y}$ | 4.1c |
|  | $l\left( \left\{ I_{o_{y}} \right\} \vert\theta\right)=\sum_{\boldsymbol{a}=\mathbf{1}}^{\boldsymbol{A}} \sum_{Y=1}^{Y} log\left[ \varphi_{N}\left( \frac{I_{o_{y}}-I_{y}}{\sigma_{I}} \right) \right]$ | 4.2c |
| Spawning Stock Biomass | ${SSB}_{y}=\sum_{a=1}^{A} N_{a,y}W_{a,y}P_{a,y}$ | 5.1c |


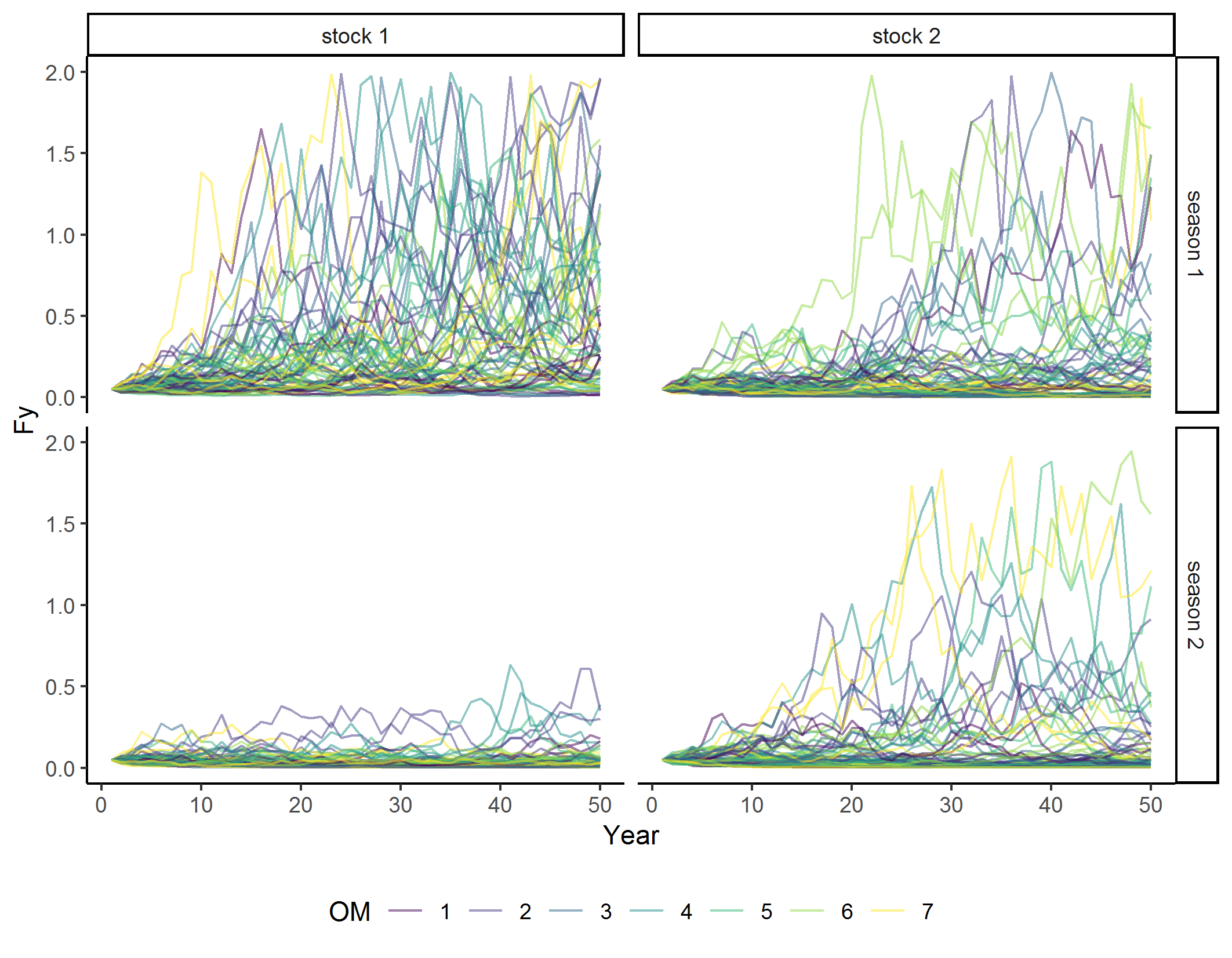


Figure A. Pseudo-Fy by stock, season and operating model (OM) for 10 random simulations of each OM.


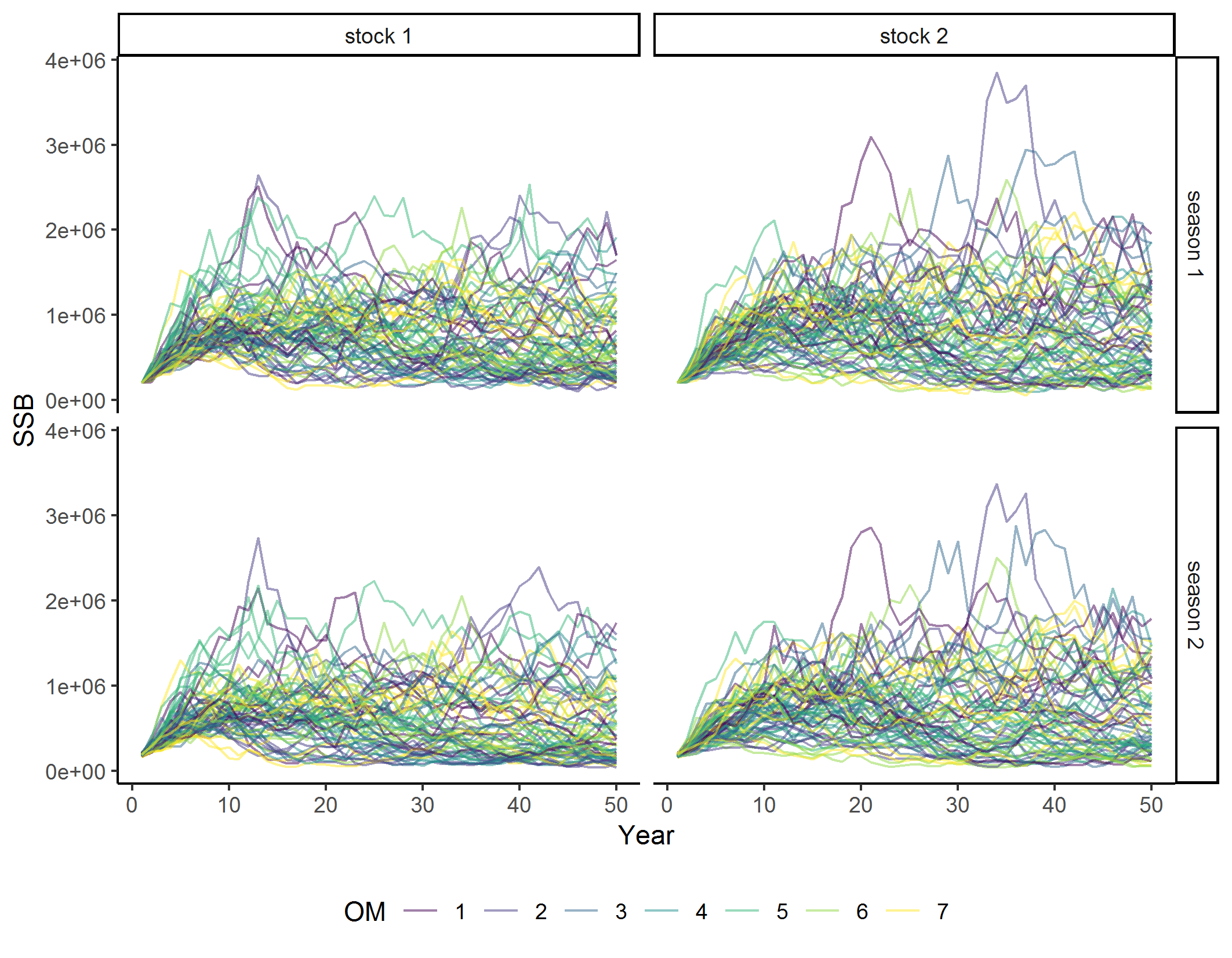


Figure B. Pseudo-SSB by stock, season and operating model (OM) for 10 random simulations of each OM.


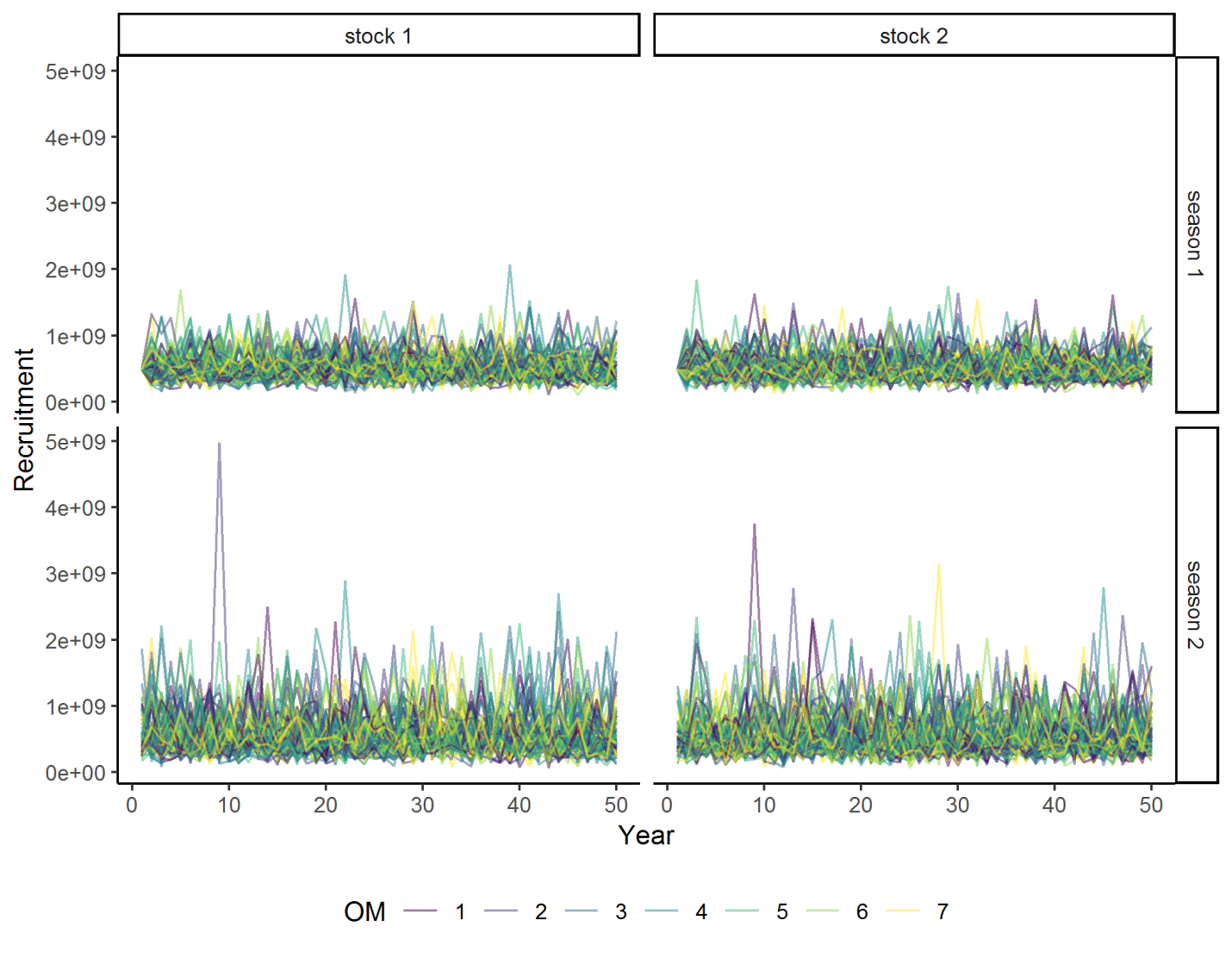


Figure C. and C Pseudo-recruitment by stock, season and operating model (OM) for 10 random simulations of each OM.


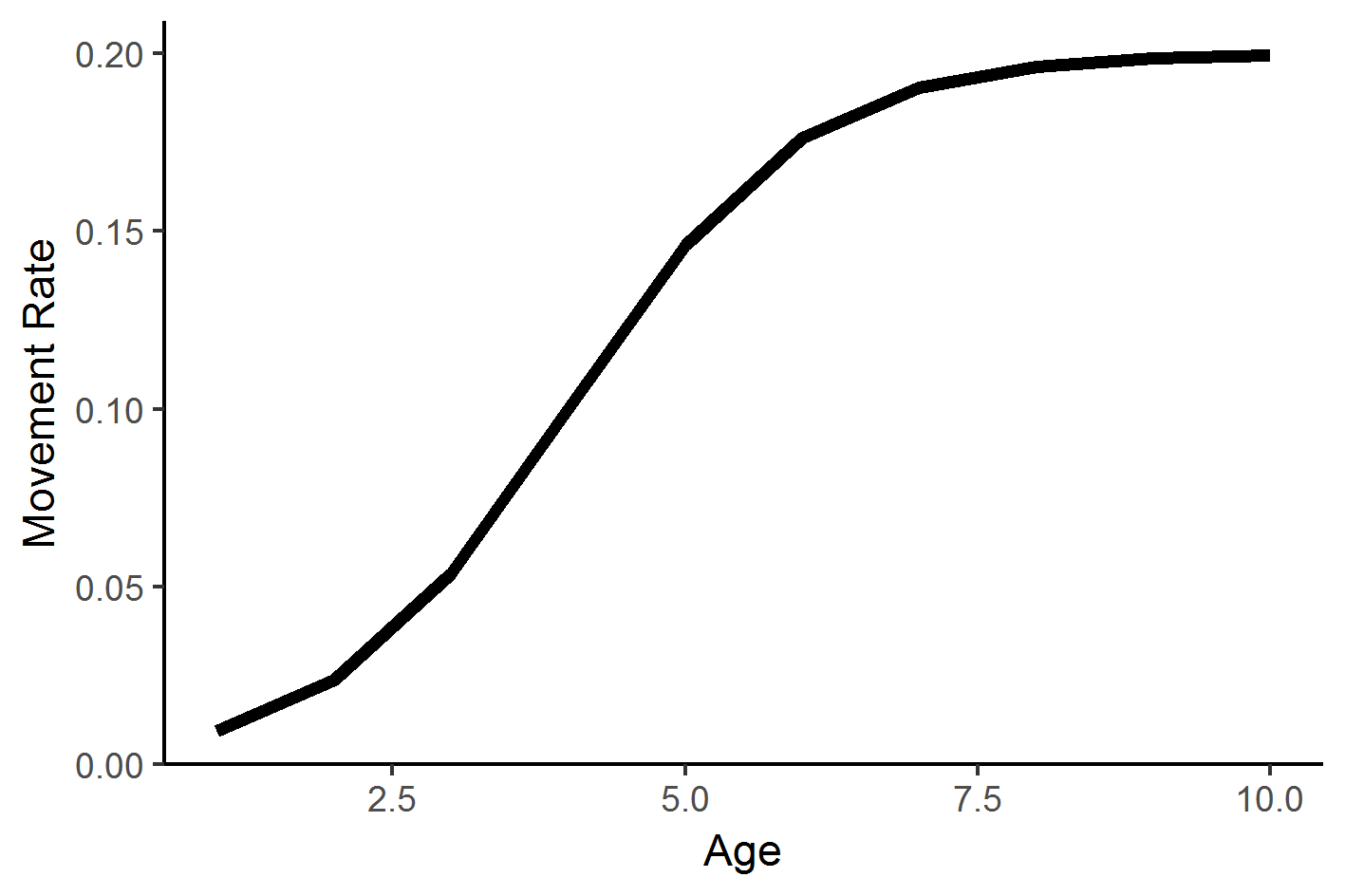


Figure D. Simulated age-dependent movement rate (operating model 6).


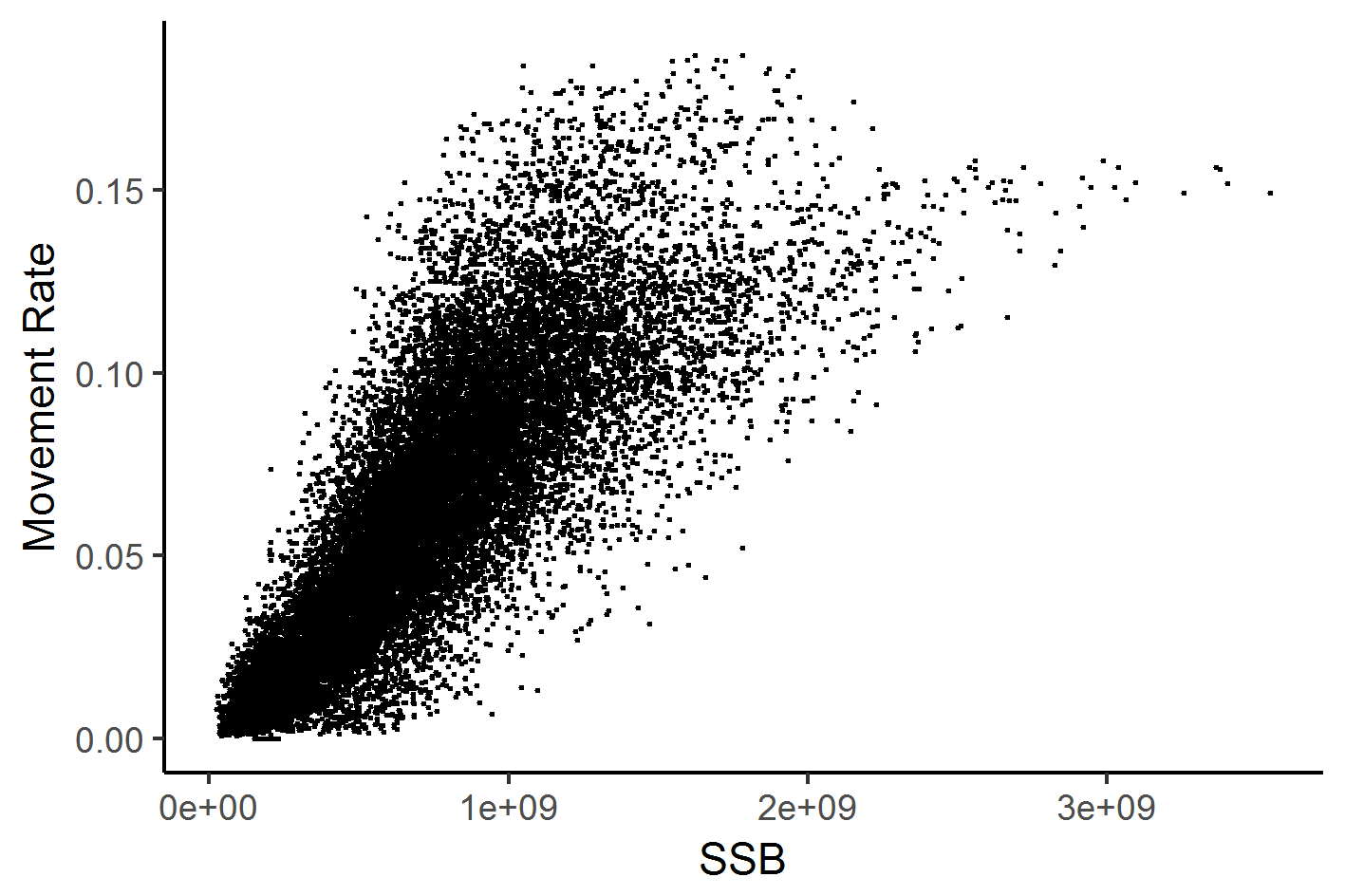


Figure E. Simulated density-dependent movement rates, both spawning components combined (operating model 7).


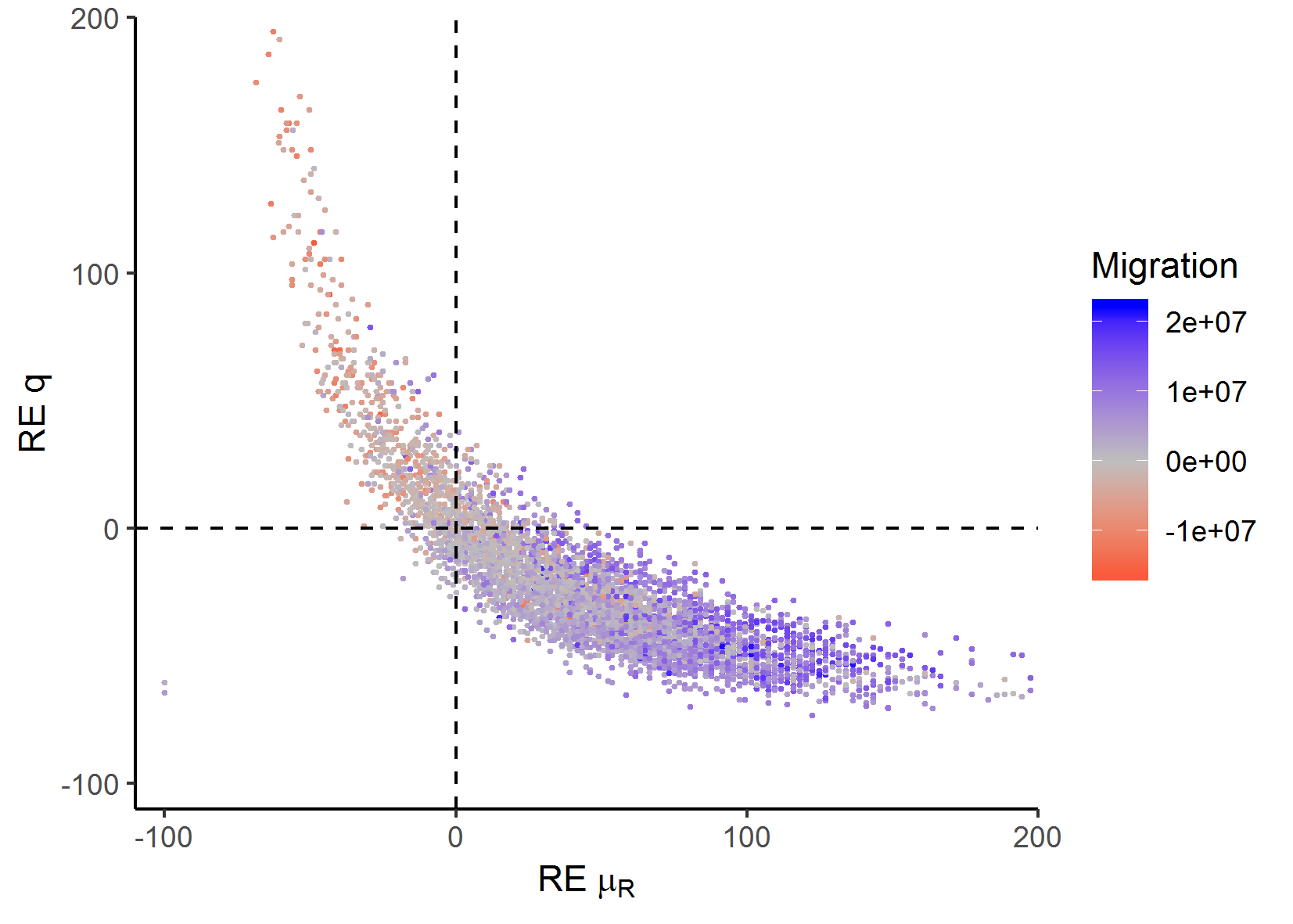


Figure F. Relationship between relative error (RE) in estimated parameters *q* (catchability) and $\mu_{R}$ (average recruitment), in function of average migration (taken over all years *y* and periods *p*) to the spawning component (blue = immigration, red = emigration). All three standard estimation models considering a spawning component were included (‘spawning component’, ‘spawning component with ignored catch’ and ‘spawning component with censored catch’), as well as all operating models presuming connectivity (operating models 2-7).
